# The Pioneer Advantage: Filling the blank spots on the map of genome diversity in Europe

**DOI:** 10.1093/gigascience/giac081

**Published:** 2022-09-09

**Authors:** Taras K Oleksyk, Walter W Wolfsberger, Khrystyna Schubelka, Serghei Mangul, Stephen J O'Brien

**Affiliations:** Uzhhorod National University, Uzhhorod, 88000, Ukraine; Oakland University, Department of Biological Sciences, Rochester, 48309 MI 48309-4479, USA; Oakland University, Department of Biological Sciences, Rochester, 48309 MI 48309-4479, USA; Oakland University, Department of Biological Sciences, Rochester, 48309 MI 48309-4479, USA; University of Southern California, USC School of Pharmacy, Los Angeles, CA 90089, USA; Nova Southeastern University, Halmos College of Natural Sciences and Oceanography, Fort Lauderdale, FL 33314, USA

**Keywords:** genomes, Europe, Ukraine, Russia, genome diversity, whole-genome sequencing, variants, genome project, genotyping, genome deserts

## Abstract

Documenting genome diversity is important for the local biomedical communities and instrumental in developing precision and personalized medicine. Currently, tens of thousands of whole-genome sequences from Europe are publicly available, but most of these represent populations of developed countries of Europe. The uneven distribution of the available data is further impaired by the lack of data sharing. Recent whole-genome studies in Eastern Europe, one in Ukraine and one in Russia, demonstrated that local genome diversity and population structure from Eastern Europe historically had not been fully represented. An unexpected wealth of genomic variation uncovered in these studies was not so much a consequence of high variation within their population, but rather due to the “pioneer advantage.” We discovered more variants because we were the first to prospect in the Eastern European genome pool. This simple comparison underscores the importance of removing the remaining geographic genome deserts from the rest of the world map of the human genome diversity.

It has been more than two decades since data of the first human genome project (HGP) were publicly released [1, 2], leading to a revolution in biomedical research. Evaluating torrents of data coming from sequencing enabled a genomic-based approach to study human health, disease, and natural history in an evolutionary context. After the HGP established the baseline for understanding common genetic variation, the analysis of genomic diversity discovered from comparing genomes of different species, multiple individuals, and across diverse populations worldwide led to the effective annotation of clinically relevant variants essential in understanding disease origin, health risks, drug sensitivity, and the promise and the perspectives of personalized medicine.

At this first stage of mapping global genome diversity, efforts were led by the global consortia of scientists who collaborated to discover and classify genome variation across the globe: Human Genome Diversity Panel (HGDP) and the 1,000 Genomes (G1K) project [3, 4] represented a monumental effort of the international community that focused on creating a comprehensive genetic diversity map of humankind. However, after the initial success, this concerted effort seems to have dissipated, leaving many blank spots, missing many local and rare variants critically important for characterization of human diversity. In the second stage of mapping worldwide genome diversity, national projects replaced the global surveys to serve as a major reference resource for human genetic variation and to provide locally based annotation of disease variants. National genome projects were supported by country governments [5, 6], international collaborations [7], and/or groups of enthusiasts [8]. These efforts continued to contribute, without the unified systematic global strategy. The projects provide an unequal geographic and population coverage and thus a fractured picture of the genome diversity across the continents.

Geographic genome surveys across populations supported the earlier conclusions of population structure, when in the 1990s, Luca Cavalli-Sforza identified 5 major clinal patterns throughout Europe [9]. While the exact distribution of these clines continues to be debated and redrawn, the population genetic structure of Europe is undoubtedly real, and similar patterns have continued to be found in more recent studies. Increasing numbers of autosomal single-nucleotide polymorphisms (SNPs; from 9,000 to 300,000) affirmed strong continent-wide correlation between geography and genetic distance [10–12]. Early surveys did not include Northeastern and Eastern Europe. Then, studies from Finland, Estonia, and the Komi Republic (Russia) showed distinct genetic diversity in Northeastern Europe, associated with the Uralic language family [13, 14]. This analysis of the European population eventually displayed a phylogeographic patterning, further underscoring the importance of local genome variation for biomedical studies [12, 15].

As of July 2022 at least 3,089 whole-genome sequences from different continental European populations have become publicly available in addition to the 2,638 genomes sequenced and publicly released by the research groups in Iceland [16] and 204,109 in the United Kingdom [17] (Fig. 1; Table 1; Supplementary Table S1). The distribution of the available genomes is still partially explained by the initial efforts by the HGDP and the G1K projects [3, 4], but a quick look at the map of Europe is enough to see that most of the data in these genome projects, as in the genotyping projects before, represent the populations of the United Kingdom and technologically advanced countries of the European Union (Fig. 1), while the diversity within many countries in Eastern Europe is represented by a handful of genomes each. The HGDP and the G1K projects followed sampling schemes that were biased by the geographic composition of the consortia and sample availability and left many important regions of genetic diversity unexplored [18]. Some of these regions will be eventually addressed by the Genome of Europe initiative that aims to build a European network of national genomic reference cohorts of at least 500,000 European citizens selected to be representative of the European population [16]. This ambitious and worthwhile goal is welcome but has not yet been released in 2022. Other projects like the Personal Genome Project may also be useful given that they carry geographical context [19]. However, as genome sequencing shifted from the international consortia to the national projects, the intrinsic bias in the distribution of human genome data available from Europe remained [20]. Table 1 incorporates the current status of the geographically referenced whole-genome data in Europe.

**Figure 1.**
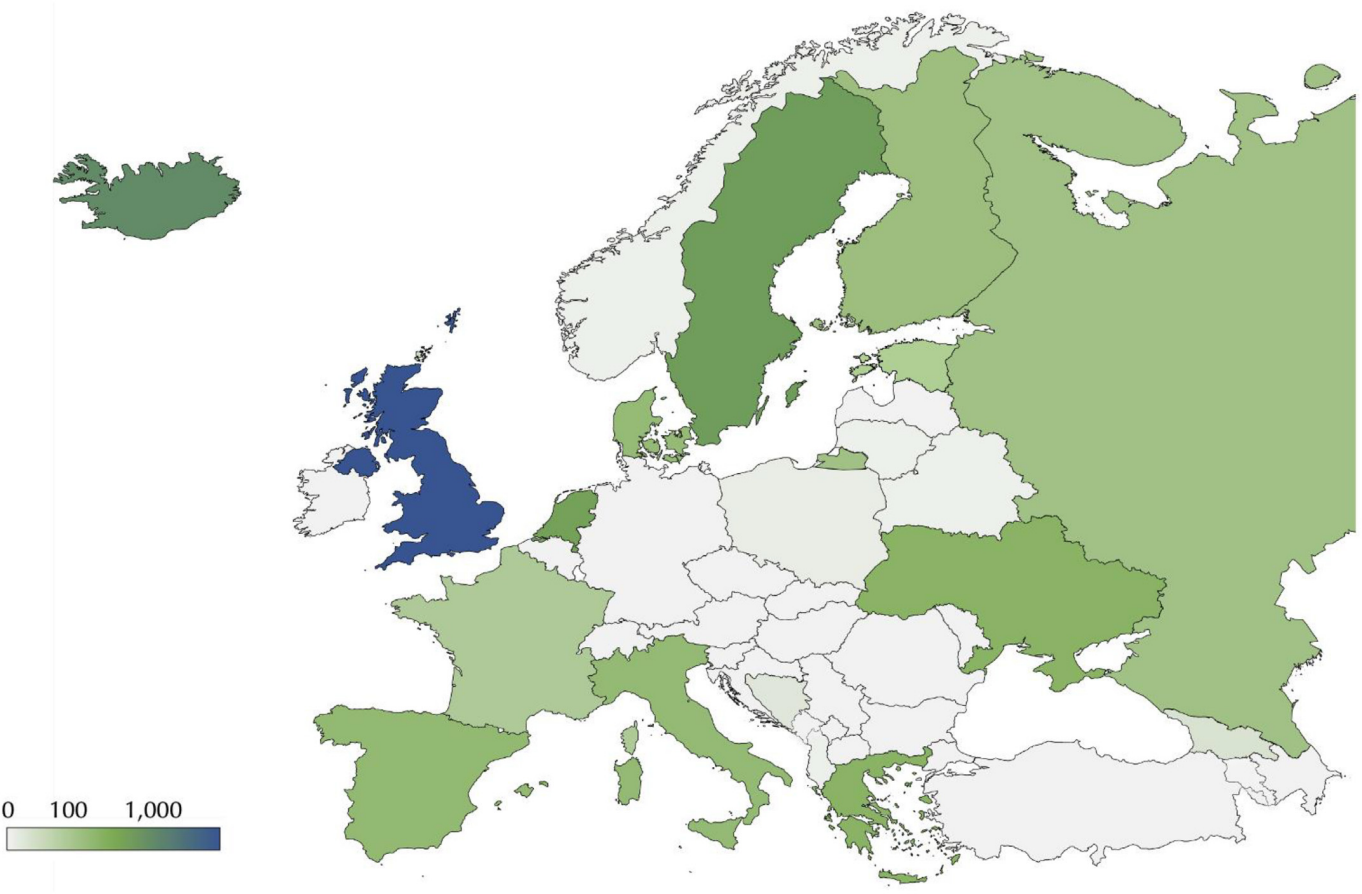
Public availability of whole-genome sequences in Europe. Numbers that represent total sample sizes for each country, geographic subpopulations, and ethnic minorities and numbers of individuals for each subpopulation/study are shown in Table 1. Links to the open data for each study summarized in this table are provided in Supplementary Table S1.

**Table 1.** Sequences of individual genomes available in Europe. The datasets are classified by source countries, and the total number of samples per country is given. Within each country, genomes may be derived from several independent studies that represent the population of the country, geographic subpopulations, and ethnic minorities, and numbers of individuals for each subpopulation/study are listed in the last column. Links to the open data for each study summarized in this table, including references, databases, and links to the studies, are provided in Supplementary Table S1. Ethnic populations and subpopulations within each country are shown in italics.

Country (population)	Total # samples per country	# Populations/studies per country	*Population or subpopulation names*	# Subpopulations/ studies	# Samples per subpopulation/study
Albania	4	*2*			
			*Albanians*	2	4
Azerbaijan	*2*	*1*			
			*Azeri*	1	2
Belarus	4	*1*			
			*Belarusians*	1	4
Bosnia-Herzegovina	7	2			
			*Croats*	1	4
			*Roma*	1	3
Bulgaria	2	1			
			*Bulgarians*	1	2
Czechia	1	1			
			*Czechi*	1	1
Denmark	150	1			
			*Danes*	1	150
Estonia	8	2			
			*Estonians*	2	8
Finland	113	4			
			*Finnish*	4	113
France	51	4			
			*Basques*	2	24
			*French*	2	27
Georgia	9	4			
			*Abkhazians*	2	5
			*Georgians*	2	4
Germany	3	1			
			*Germans*	1	3
Greece	254	3			
			*Cretans*	2	252
			*Mainland Greeks*	1	2
Hungary	3	2			
			*Hungarians*	2	3
Iceland	2,638	2			
			*Icelanders*	2	2,638
Italy	159	7			
			*Italians*	5	132
			*Sardinians*	2	27
Latvia	3	1			
			*Latvians*	1	3
Lithuania	4	2			
			*Lithuanians*	2	4
Moldova	2	1			
			*Moldovans*	1	2
Netherlands	769	1			
			*Dutch*	1	769
Norway	4	2			
			*Finnish*	1	3
			*Norwegians*	1	1
Orkney Islands	15	2			
			*Orcadians*	2	15
Poland	5	2			
			*Poles*	2	5
Russia*	178	34			
			*Adygeis*	2	17
			*Avars*	1	3
			*Azerbaijanis*	1	1
			*Balkars*	1	3
			*Bashkirs*	1	5
			*Chechen*	1	1
			*Circassians*	1	3
			*Ingrians*	1	3
			*Kabardins*	1	4
			*Karelians*	1	3
			*Khantys*	1	3
			*Komis*	1	2
			*Kryashen-Tatars*	1	3
			*Kuban-Cossacks*	1	2
			*Kumyks*	1	3
			*Lezgins*	2	6
			*Mansis*	2	5
			*Maris*	1	4
			*Mishar-Tatars*	1	1
			*Mordvins*	1	3
			*North-Ossetians*	2	4
			Russians*	7	92
			*Tabasarans*	1	3
			*Vepsas*	1	4
Spain	164	2			
			*Spanish*	2	164
Sweden	1,002	2			
			*Swedes*	2	1,002
Turkey	*2*	*1*			
			*Turks*	2	1
Ukraine	257	7			
			*Cossacks*	1	2
			*Hungarians*	1	1
			*Ukrainians*	5	254
United Kingdom	204,109	3			
			*British*	3	204,107
			*English*	1	2
Total	9,917	96			209,836

*Only the populations native to the European part of the Russian Federation are represented in this survey.

Multiple types of efforts to aggregate current known variation are under way. To cover the highest number of samples and include projects with varying data release strategies, first the Exome Aggregation Consortium (ExAC) and then the Genome Aggregation Database (gnomAD) omitted the individual-level information. Today, gnomAD has provided publicly available and accessible variation summaries from as many as 76,156 individual genomes of various projects across the globe [21, 22]. These datasets are easy to handle and to interpret and can be useful for comparative analysis and identification of novel variation, but they are not well suited for population analysis on the smaller scale. On the other hand, the Database of Genotypes and Phenotypes provides resources like the individual variants and an extensive amount of phenotypical information per sample but requires additional bioinformatic expertise to handle [23]. Often, the nature of these data means that data access is controlled and requires an application with appropriate justification. To ensure data security, specialized instruments and tokens are often needed to reach the data. These necessary precautions can create delays or outright limit a research group in its ability to reach the data, given a lack of technical expertise.

Recently, analysis of the data from whole-genome studies, one in Ukraine [8] and one in Russia [24], clearly demonstrated that intrinsic genomic diversity from Eastern Europe had been poorly represented. The SNP allele frequency differences are large enough to provide previously unknown dimensions of population structure. For instance, Zhernakova et al. [24] identified 5 distinct phylogeographic population partitions from east to west across the 11 time zones of the Russian Federation. They also reported important genetic differences between ethnic Russian populations and their neighbors. The principal component analysis of Russian subpopulations from Pskov and Novgorod in the European part of Russia compared with other populations of Europe and Asia (264 study participants) demonstrated genetic distinctiveness as great or greater than differences between populations of neighboring Finns, Swedes, and Estonians [24]. A subsequent analysis of Ukrainian genomes showed that the Ukrainian cluster was distinct from publicly available European populations or publicly available genomes from subpopulations of ethnic Russians in the European part of Russia [3,8].

These 2 studies discovered millions of mutations, many of which were previously not described (478,000 in Ukraine [8] and approximately 300,000 in 2 populations from the European Russia [24]) and reported major differences in frequencies of medically related alleles between Eastern Europe and the rest of the continent. This wealth of genomic variation uncovered in Ukraine and, to the lesser extent, in Russia was not due to the particularly high variation within the Ukrainian population but to the absence of sampling variation from the countries around Ukraine.

The effect observed is due to what is known in the business and marketing world as a “first-mover” or “pioneer advantage” [25]. Since the Ukrainian population was surrounded by unexplored genome deserts, the study of fewer than 100 Ukrainian genomes uncovered 478,000 novel genomic SNPs that have never been previously registered in the Genome Aggregation Database [26]. This number is huge, even in comparison to the most genetically diverse populations in sub-Saharan Africa, where the addition of new genomes from 426 people across 50 ethnolinguistic groups to the existing databases revealed approximately 3 million variants [27]. This simple comparison underscores the importance of complete information about the global extent of genome variation and removing remaining genome deserts from the rest of the world map.

Understanding all geographic dimensions of genome diversity in Europe is crucial for the local biomedical community to use local genomics data instead of extrapolating results from genome projects in other countries. The importance of sequence data from multiple populations cannot be underestimated, given their unique histories of drift, selection, migration, admixture, and socioeconomic structures. Therefore, we suggest that every country needs its own national genome database to inform regionally relevant and objective public health policies. There is still a lot of important variation to be discovered, and it needs to be made public to provide the informational framework for the biomedical research to follow.

Currently, the largest genome representation in Europe is in the United Kingdom [5] due to a well-funded national project, including more that 200,000 genomes publicly available for analysis. Countries like Iceland (2,638), the Netherlands (769), Italy (159), Spain (164), and Sweden (1,002) also shared large sequence databases with the scientific community (Table 1). Still, many countries in Europe remain underrepresented. Aside from Belgium, Portugal, and Switzerland, most of these underrepresented countries are in Eastern Europe. Poland has recently started its national genome project that will soon contribute thousands of genomes to the public domain [28]. Austria, Croatia, Montenegro, North Macedonia, Romania, Serbia, and Slovakia have no representation in the public genome databases yet, and several other countries such as Bosnia-Herzegovina, Bulgaria, Belarus, Czechia, Hungary, Lithuania, Latvia, and Moldova have only a handful genomes included in international projects [4, 7, 29].

Funding for the national genome projects has come from various sources and often combines private and public sources. Unfortunately, successful genomic initiatives rarely come from countries from smaller economies. Ukraine is an outlier in this category (an Eastern European country with a relatively small economy with 254 genomes available), due to a successful collaboration strategy. Lacking support of the Ukrainian government, samples became available through international collaborations with BGI (formerly the Beijing Genomics Institute) as well as the National Institutes of Health (USA), specifically to help fill in the genome desert in that country [8]. This approach can be replicated if the principal roadblock to a national genome project is the lack of local funding in countries with smaller economies.

The national genomic projects offer an effective platform for training genome scientists and bioinformaticians at the national level. Research teams involved in national genomic projects should be multidisciplinary and interinstitutional and include policy makers, lawyers, data scientists, and human geneticists. There is a critical need for involving experts in the humanities, especially those who understand relevant ethical and social issues. Given this complexity, international collaborations can be very helpful and can be defined from the beginning of the project, not only for writing the collection protocols and providing sequencing platforms but also for serving the ultimate objectives—improving public health in each country.

Politics matters in these efforts and not always for the good. The Genome Russia project was conceived as a platform that would bring together scientists from across the Russian Federation with international collaborations from across the globe (http://genomerussia.spbu.ru/) [6, 18]. The hope was that this project would include a program to train bioinformatics experts who could carry on the torch for the next generation of genome studies. After the initial success in building a consortium and a first analysis/data publication [24], public access to the genome data was initially approved by NCBI, then abruptly retracted by direct order of Russian authorities after the paper was published. The 2022 hostilities in Ukraine from Russian invaders bode rather poorly for immediate remedy of these issues. The publication of Ukrainian genomes was met with hostility by the Russian authorities, who demanded the retraction of the manuscript, first the preprint from *bioRxiv* and then the paper from *GigaScience* [30]. Genome diversity of the Ukraine paper was ultimately published, and all the data (except the Genome Russia genomes) were released publicly over the Russian objections and presure.

As stipulated in the original proposal and development documents for the Genome Russia and cooperative agreements for the Genome Diversity in Ukraine projects, the principal goal of these efforts was to provide open access of all sequence data SNP annotation and other genome features, so that these could join other international genome sequence consortia releasers [6, 8, 18, 24]. The design and even the informed consent protocols for both of these projects were actually modeled after the 1,000 Genomes Project, which had been vetted by the world's experts on human genome ethics. The original intention and promise was to join and augment the catalogue of genomes in the 1,000 Genomes Project as well as the SNP annotations to complement ExAc [22], gnomAd [21], Gene Mutation Database (HGMD), and HGMD-DM (disease-causing mutations) [31]. The political shuttering of open release by Russian authorities would ultimately cost all these important projects until release was remedied. This ill-advised suspension of open release was further exacerbated by the 2022 February 24 invasion of Ukraine ordered by Russian President Putin.

Researchers can still retrieve ethnic Russian genomes scattered in small batches across other publications, as well as from modest representation of multiple non-Russian ethnic minorities who make up almost half of the genomes still available from the European part of the Russian Federation. From the European part of Russia, genomes from 23 ethnically distinct indigenous populations are available, such as Ossetians, Tatars, Chechens, Komis, Bashkirs, Mordvins, and others, in addition to 7 subpopulations of ethnic Russians (Table 1).

The uneven distribution of the genome data in Europe is further exacerbated by widespread lack of data sharing in the scientific community. While commercial companies are compiling but restricting massive data among themselves, the genome-wide sequence data from ancestry testing and diagnostic sequencing [32] are difficult or impossible to retrieve [24,33]. This is usually justified by the possibility that, even when the databases are completely anonymous, it is technically possible to identify participating individuals with additional genotype information [34]. Therefore, publishing individual genome data needs appropriate levels of informed consent that require a combination of technical and societal stipulations within the context in which the data are released. There remains a stunning lack of consensus or compelling legal precedents involving ownership or open release of biomedical materials and derivative data [35]. Different published studies (genomes, GWAS (Genome Wide association Study), and others) have been collected under widely diverse levels of informed consent agreed on by the study participants. All this ambiguity compels many in management/privacy positions to simply deny access to both samples and data, a serious problem that may be getting worse.

We emphasize the importance of public access to the data that is consented and open. The best practice, pioneered by the G1K consortium, was to deposit the collected genome sequences, informed consent details, and the accompanying data into an international database that could serve as a valuable resource for the researchers worldwide, while providing security and protecting the interests of participating individuals and the communities they represent [4]. Publicly available genome data generated from the general population of the country have a vital role to unlock the capacities of genomic-based personal medicine for residents of a given county and benefit everyone.

## Abbreviations

ExAC: Exome Aggregation Consortium; G1K: 1,000 Genomes Project; gnomAD: Genome Aggregation Database; HGDP: Human Genome Diversity Panel; HGP: Human Genome Project; SNP: single-nucleotide polymorphism.

## Funding

TKO, WW and KS were supported in part by 2SOFT/1.2/48 project "Partnership for Genomic Research in Ukraine and Romania" by the Joint Operational Programme Romania-Ukraine, through the European Neighbourhood Instrument (ENI).

## Data Availability

Links to all the data mentioned in this article are available in Supplemental Table S1.

## Competing Interests

The authors declare that they have no competing interests.

## Authors' Contributions

T.K.O. has written the first draft. W.W.W. and K.S. prepared and analyzed the data. S.M. and S.J.O. contributed to the original ideas and writing and final editing of the manuscript.

## Supplementary Material

giac081_GIGA-D-22-00114_Original_Submission

giac081_GIGA-D-22-00114_Revision_1

giac081_Response_to_Reviewer_Comments_Original_Submission

giac081_Reviewer_1_Report_Original_Submission Aniko Sabo -- 7/18/2022 Reviewed

giac081_Supplemental_File
