# Supplementary material for: The Pioneer Advantage: Filling the blank spots on the map of genome diversity in Europe
Source: Gigascience. 2022 Sep 9;11:giac081. doi: 10.1093/gigascience/giac081 (PMC9463063; doi:10.1093/gigascience/giac081)
Supplement: giac081_GIGA-D-22-00114_Original_Submission [file giac081_giga-d-22-00114_original_submission.pdf]

GigaScience

The Pioneer’s Advantage: Filling the Blank Spots On the Map of Genome Diversity in Europe

--Manuscript Draft--

**Manuscript Number:**	GIGA-D-22-00114
**Full Title:**	The Pioneer’s Advantage: Filling the Blank Spots On the Map of Genome Diversity in Europe
**Article Type:**	Review
**Funding Information:**	
**Abstract:**	Documenting genome diversity is important for the local biomedical communities and instrumental in developing precision and personalized medicine. Currently, near ten thousand whole genome sequences from Europe are publicly available, but most of these represent populations of developed countries of Europe. The uneven distribution of the available data is further impaired by the lack of data sharing. Recent whole-genome studies in Eastern Europe, one in Ukraine and one in Russia, demonstrated that local genome diversity and population structure from Eastern Europe historically had not been fully represented. An unexpected wealth of genomic variation uncovered in these studies was not so much a consequence of high variation within their population, but rather due to the “pioneer advantage”. We discovered more variants because we were the first to prospect in the Eastern European genome pool. This simple comparison underscores the importance of removing the remaining geographic genome deserts from the rest of the world map of the human genome diversity.
**Corresponding Author:**	Taras K Oleksyk, Ph.D.<br>Oakland University<br>Rochester, MI UNITED STATES
**Corresponding Author Secondary Information:**	
**Corresponding Author's Institution:**	Oakland University
**Corresponding Author's Secondary Institution:**	
**First Author:**	Taras K Oleksyk, Ph.D.
**First Author Secondary Information:**	
**Order of Authors:**	Taras K Oleksyk, Ph.D.
	Walter W Wolfsberger, MS
	Serghei Mangul, Ph.D.
	Stephen J O'Brien, Ph.D.
**Order of Authors Secondary Information:**	
**Additional Information:**	
**Question**	**Response**
Are you submitting this manuscript to a special series or article collection?	No
**Experimental design and statistics**<br><br>Full details of the experimental design and statistical methods used should be given in the Methods section, as detailed in our Minimum Standards Reporting Checklist.	No

Information essential to interpreting the data presented should be made available in the figure legends.<br><br>Have you included all the information requested in your manuscript?	
If not, please give reasons for any omissions below.<br><br>as follow-up to "**Experimental design and statistics**<br><br>Full details of the experimental design and statistical methods used should be given in the Methods section, as detailed in our Minimum Standards Reporting Checklist. Information essential to interpreting the data presented should be made available in the figure legends.<br><br>Have you included all the information requested in your manuscript?<br><br>"	In this commentary we do no use our own data
**Resources**<br><br>A description of all resources used, including antibodies, cell lines, animals and software tools, with enough information to allow them to be uniquely identified, should be included in the Methods section. Authors are strongly encouraged to cite Research Resource Identifiers (RRIDs) for antibodies, model organisms and tools, where possible.<br><br>Have you included the information requested as detailed in our Minimum Standards Reporting Checklist?	No
If not, please give reasons for any omissions below.	We do not use any such resources

Powered by Editorial Manager® and ProduXion Manager® from Aries Systems Corporation

as follow-up to "**Resources**<br><br>A description of all resources used, including antibodies, cell lines, animals and software tools, with enough information to allow them to be uniquely identified, should be included in the Methods section. Authors are strongly encouraged to cite Research Resource Identifiers (RRIDs) for antibodies, model organisms and tools, where possible.<br><br>Have you included the information requested as detailed in our Minimum Standards Reporting Checklist?<br><br>"	
**Availability of data and materials**<br><br>All datasets and code on which the conclusions of the paper rely must be either included in your submission or deposited in publicly available repositories (where available and ethically appropriate), referencing such data using a unique identifier in the references and in the "Availability of Data and Materials" section of your manuscript.<br><br>Have you have met the above requirement as detailed in our Minimum Standards Reporting Checklist?	Yes

Powered by Editorial Manager® and ProduXion Manager® from Aries Systems Corporation

Manuscript

Click here to access/download;Manuscript;Pioneers advantage.06.11.2022.v6.TKO.docx

Review

The Pioneer's Advantage:

Filling the Blank Spots On the Map of Genome Diversity in Europe

Taras K. Oleksyk (ORCID ID 0000-0002-8148-3918, Oakland University, MI and Uzhhorod National University, Ukraine)

Walter W. Wolfsberger (ORCID ID 0000-0003-0980-645X,

Oakland University, MI)

Serghei Mangul (ORCID ID 0000-0003-4770-3443,

University of Southern California, CA)

Stephen J. O'Brien (ORCID ID 0000-0001-7353-8301,

Nova Southeastern University, FL)

Corresponding author: Taras K Oleksyk, Department of Biological Sciences, Oakland University

Dodge Hall Rm 367, 118 Library Dr., Rochester, MI 48309-4479

Oleksyk@oakland.edu

Abstract

Documenting genome diversity is important for the local biomedical communities and instrumental in developing precision and personalized medicine. Currently, near ten thousand whole genome sequences from Europe are publicly available, but most of these represent populations of developed countries of Europe. The uneven distribution of the available data is further impaired by the lack of data sharing. Recent whole-genome studies in Eastern Europe, one in Ukraine and one in Russia, demonstrated that local genome diversity and population structure from Eastern Europe historically had not been fully represented. An unexpected

wealth of genomic variation uncovered in these studies was not so much a consequence of high variation within their population, but rather due to the “pioneer advantage”. We discovered more variants because we were the first to prospect in the Eastern European genome pool. This simple comparison underscores the importance of removing the remaining geographic genome deserts fromm the rest of the world map of the human genome diversity.

Keywords

Genomes; Eastern Europe; Ukraine; Russia; genome diversity; phylogeography

Main Text

It has been more than two decades since data of the first **human genome project (HGP)** was publicly released [1,2], leading to a revolution in biomedical research. Evaluating torrents of data coming from sequencing enabled genomic-based approach to study human health, disease and natural history in an evolutionary context. After the HGP established the baseline for understanding common genetic variation, the analysis of genomic diversity discovered from comparing genomes of different species, multiple individuals, and across diverse populations worldwide led to the effective annotation of medically related variants essential in understanding disease origin, health risks, drug sensitivity, the promise and the perspectives of personalized medicine.

At this **first stage of mapping global genome diversity**, efforts were led by the global consortia of scientists that collaborated to discover and classify genome variation across the globe: Human Genome Diversity Panel (HGDP) and the 1,000 Genomes (G1K) project [3,4] represented a monumental effort of the international community that focused on creating a comprehensive genetic diversity map of the humankind. However, after the initial success, this concerted effort seems to have dissipated leaving many blank spots, missing many local and rare variants critically important for characterization of human diversity. In the **second stage of mapping worldwide genome diversity**, national projects replaced the global surveys to serve as a major reference resource for human genetic variation and to provide locally based annotation of disease variants. National genome projects were supported by country governments [5,6], international collaborations [7] and/or groups of enthusiasts [8]. These efforts continued to

contribute, without the unified systematic global strategy. The projects provide an unequal geographic and population coverage and thus a fractured picture of the genome diversity across the continents.

Geographic genome surveys across populations supported the earlier conclusions **of population structure**, when in the 1990s Luca Cavalli-Sforza identified five major clinal patterns throughout Europe [9]. While the exact distribution of these clines continues to be debated and redrawn, population genetic structure of Europe is undoubtedly real, and similar patterns have continued to be found in more recent studies. Increasing numbers of autosomal Single Nucleotide Polymorphisms (SNPs; from 9k to 300K) affirmed strong continent-wide correlation between geography and genetic distance [10–12]. Early surveys did not include Northeastern and Eastern Europe. Then studies from Finland, Estonia and the Komi Republic (Russia) showed distinct genetic diversity in Northeastern Europe, associated with the Uralic language family [13,14]. This analysis of European population eventually displayed a phylogeographic patterning, further underscoring the importance of local genome variation for biomedical studies [12,15].

In the last decade since the founding of *GigaScience*, and as of July 2022, at least **3,089** whole genome sequences from different continental European populations have become publicly available in addition to the 2,638 genomes sequenced and publicly released by the research groups in Iceland [16] and 204,109 in the UK [17] **(Figure 1; Table 1; Table S1).** The distribution of the available genomes is still partially explained by the initial efforts by the HGDP and the G1K projects [3,4], but a quick look at the

map of Europe is enough to see that most of the data in these genome projects, as in the genotyping projects before, represents the populations of the UK and technologically advanced countries of the European Union (**Figure 1**), while the diversity within many countries in Eastern Europe are represented by a handful of genomes each. The HGDP and the G1K projects followed sampling schemes that were biased by the geographic composition of the consortia and sample availability, and left many important regions of genetic diversity unexplored [18]. Some of these regions will be eventually addressed by the Genome of Europe (GoE) initiative that aims to build a European network of national genomic reference cohorts of at least 500,000 European citizens selected to be representative of the European population [16]. This ambitious and worthwhile goal is welcome but has not yet been released in 2022. Other projects like the Personal Genome Project (PGP) may also be useful given that they carry geographical context [19]. However, as genome sequencing shifted from the international consortia to the national projects, the intrinsic bias in the distribution of human genome data available from Europe remained [20]. **Table 1** incorporates the current status of the geographically referenced whole genome data in Europe.

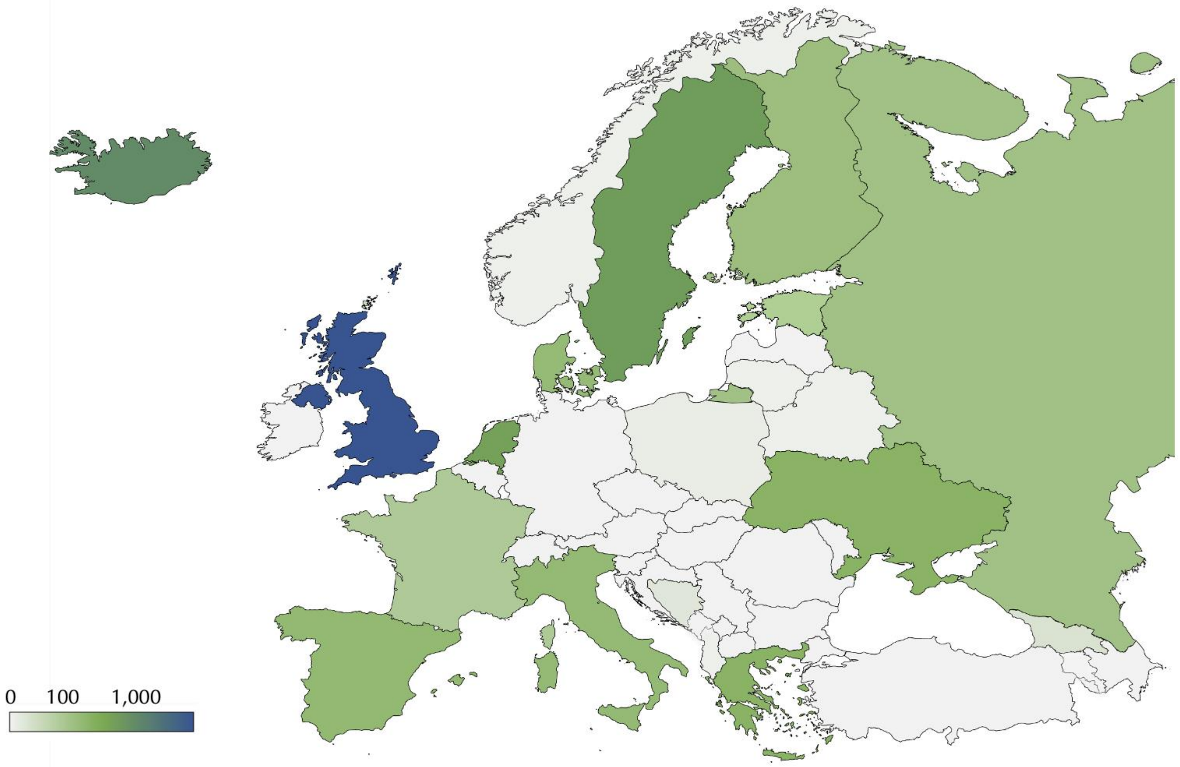

Figure 1. Public availability of whole genome sequences in Europe. Numbers that represent total sample sizes for each country, geographic sub-populations, and ethnic minorities, and numbers of individuals for each sub-population/study are shown in **Table 1**. Links to the open data for each study summarized in this table are provided in **Table S1**.

Table 1. Sequences of individual genomes available in Europe. The datasets are classified by source countries, and the total number of samples per country is given. Within each country genomes may be derived from be several independent studies that represent the population of the country, geographic sub-populations, and ethnic minorities, and numbers of individuals for each sub-population/study. is listed in the last column. Links to the open data for each study summarized in this table, including references, databases, and links to the studies are provided in **Table S1**.

**Country (population)**	**Total # samples per country**	**# Populations/ Studies per country**	***Population or sub-population names***	**# Sub-populations/ Studies**	**# Samples per sub-population/study**
**Albania**	**4**	***2***			
			Albanians	2	4
**Azerbaijan**	*2*	*1*			

			Azeri	1	2
**Belarus**	**4**	***1***			
			Belarusians	1	4
**Bosnia-Herzegovina**	**7**	**2**			
			Croats	1	4
			Roma	1	3
**Bulgaria**	**2**	**1**			
			Bulgarians	1	2
**Czechia**	**1**	**1**			
			Czechi	1	1
**Denmark**	**150**	**1**			
			Danes	1	150
**Estonia**	**8**	**2**			
			Estonians	2	8
**Finland**	**113**	**4**			
			Finnish	4	113
**France**	**51**	**4**			
			Basques	2	24
			French	2	27
**Georgia**	**9**	**4**			
			Abkhazians	2	5
			Georgians	2	4
**Germany**	**3**	**1**			
			Germans	1	3
**Greece**	**254**	**3**			
			Cretans	2	252
			Mainland Greeks	1	2
**Hungary**	**3**	**2**			

			Hungarians	2	3
**Iceland**	**2,638**	**2**			
			Icelanders	2	2,638
**Italy**	**159**	**7**			
			Italians	5	132
			Sardinians	2	27
**Latvia**	**3**	**1**			
			Latvians	1	3
**Lithuania**	**4**	**2**			
			Lithuanians	2	4
**Moldova**	**2**	**1**			
			Moldovans	1	2
**Netherlands**	**769**	**1**			
			Dutch	1	769
**Norway**	**4**	**2**			
			Finnish	1	3
			Norwegians	1	1
**Orkney Islands**	**15**	**2**			
			Orcadians	2	15
**Poland**	**5**	**2**			
			Poles	2	5
**Russia**	**178**	**34**			
			Adygeis	2	17
			Avars	1	3
			Azerbaijanis	1	1
			Balkars	1	3
			Bashkirs	1	5
			Chechen	1	1
			Circassians	1	3

			Ingrians	1	3
			Kabardins	1	4
			Karelians	1	3
			Khantys	1	3
			Komis	1	2
			Kryashen-Tatars	1	3
			Kuban-Cossacks	1	2
			Kumyks	1	3
			Lezgins	2	6
			Mansis	2	5
			Maris	1	4
			Mishar-Tatars	1	1
			Mordvins	1	3
			North-Ossetians	2	4
			*Russians**	7	92
			Tabasarans	1	3
			Vepsas	1	4
**Spain**	**164**	**2**			
			Spanish	2	164
**Sweden**	**1,002**	**2**			
			Swedes	2	1,002
**Türkiye**	***2***	***1***			
			Turks	2	1
**Ukraine**	**257**	**7**			
			Cossacks	1	2
			Hungarians	1	1
			Ukrainians	5	254
**The United Kingdom**	**204,109**	**3**			

			British	3	204,107
			English	1	2
**Total**	**9,917**	**96**			**209,836**

Recently, analysis of the data from whole-genome studies, one in Ukraine [8] and one in Russia [21], clearly demonstrated that intrinsic genomic diversity from Eastern Europe had been poorly represented. The SNP allele frequency differences are large enough to provide previously unknown dimensions of population structure. For instance, Zhernakova et al. [21] identified five distinct phylogeographic population partitions from east to west across the eleven time zones of the Russian Federation. They also reported important genetic differences between ethnic Russian populations and their neighbors. The Principal Component Analysis (PCA) of Russian sub-populations from Pskov and Novgorod in the European part of Russia compared with other populations of Europe and Asia (264 study participants), demonstrated genetic distinctiveness that are as great or greater than differences between populations of neighboring Finns, Swedes and Estonians [21]. A subsequent analysis of Ukrainian genomes showed that Ukrainian cluster was distinct from publicly available European populations or publicly available genomes from sub-populations of ethnic Russians in the European part of Russia [3,8].

These two studies discovered millions of mutations many of which were previously not described (478k in Ukraine [8] and approximately 300k in two populations from the European Russia [21]) and reported major differences in frequencies of medically related alleles between Eastern Europe and the rest of the continent. This wealth of genomic

variation uncovered in Ukraine and to the lesser extent in Russia was not due to the particularly high variation within the Ukrainian population, but to the absence of sampling variation from the countries around Ukraine.

The effect observed is due to what is known in the business and marketing world as a "first-mover" or "pioneer advantage"[22]. Since the Ukrainian population was surrounded by unexplored genome deserts, the study of less than one hundred Ukrainian genomes uncovered 478,000 novel genomic SNPs that have never been previously registered in the Genome Aggregation Database [23]. This number is huge, even in comparison to the most genetically diverse populations in Sub-Saharan Africa, where the addition of new genomes from 426 people across 50 ethnolinguistic groups to the existing databases revealed approximately 3 million variants [24]. This simple comparison underscores the importance of complete information about global extent of genome variation and removing remaining genome deserts form the rest of the world map.

Understanding all geographic dimensions of genome diversity in Europe is crucial for the local biomedical community to use local genomics data instead of extrapolating results from genome projects in other countries. The importance of sequence data from multiple populations cannot be underestimated, given their unique histories of drift, selection, migration, admixture, and socioeconomic structures. Therefore, we suggest that every country needs its own national genome database to inform regionally relevant and objective public health policies. There is still a lot of important variation to be

discovered, and it needs to be made public to provide the informational framework for the biomedical research to follow.

Currently the largest genome representation in Europe is in the UK [5] due to well-funded national project, including more that 200k genomes publicly available for analysis. Countries like Iceland (2,638), the Netherlands (769), Italy (159), Spain (164), and Sweden (1002) also shared large sequence databases with the scientific community (**Table 1**). Still, many countries in Europe remain underrepresented. Aside from Belgium, Portugal and Switzerland, most of these underrepresented countries are in Eastern Europe. Poland has recently started its national genome project that will soon contribute thousands of genomes to the public domain [25]. Austria, Croatia, Montenegro, North Macedonia, Romania, Serbia, and Slovakia have no representation in the public genome databases yet, and several other countries such as Bosnia-Hercegovina, Bulgaria, Belarus, Czechia, Hungary, Lithuania, Latvia and Moldova, have only a handful genomes included in international projects [4,7,26].

Funding for the national genome projects has come from various sources, and often combines private and public sources. Unfortunately, successful genomic initiatives rarely come from countries from smaller economies. Ukraine is an outlier on this category (an Eastern European country with a relatively small economy with 254 genomes available), due to a successful collaboration strategy. Lacking support of the Ukrainian government, samples became available through international collaborations with BGI as well as the NIH, specifically to help fill in the genome desert in that country [8]. This approach can

be replicated if the principal roadblock to a national genome project is the lack of local funding in countries with smaller economies.

The national genomic projects offer an effective platform for training genome scientists and bioinformaticians at national level. Research teams involved in national genomic projects should be multidisciplinary, inter-institutional and include policy makers, lawyers, data scientists, and human geneticists. There is a critical need of involving experts in the humanities, especially those who understand relevant ethical and social issues. Given this complexity, international collaborations can be very helpful, and can be defined from the beginning of the project, not only for writing the collection protocols and provide sequencing platforms, but to serve the ultimate objectives – improving public health in each country.

Politics matters in these efforts and not always for the good. The Genome Russia project was conceived as a platform that would bring together scientists from across the Russian Federation with international collaborations from across the globe (http://genomerussia.spbu.ru/) [6,18]. The hope was that this project would include a program to train bioinformatics experts who could carry on the torch in for the next generation of genome studies. After the initial success in building a consortium, and a first analysis/data publication [21], public access to the genome data was initially approved to NCBI , then abruptly retracted by direct order of Russian authorities after the paper was published. The 2022 hostilities in Ukraine from Russian invaders bodes rather poorly for immediate remedy of these issues. The publication of Ukrainian

genomes was met with hostility by the Russian authorities who demanded the retraction of the manuscript, first the preprint from *bioRxiv* and then the paper from *GigaScience* [27]. Genome diversity of Ukraine paper was ultimately published and all the data (except the Genome Russia genomes) was released publicly over strenuous Russian objections.

Researchers can still retrieve ethnic Russian genomes scattered is small batches across other publications, as well as from modest representation of multiple non-Russian ethnic minorities that make up almost a half of genomes still available from the European part of the Russian Federation. From the European part of Russia, genomes from 23 ethnically distinct indigenous populations are available, such as Ossetians, Tatars, Chechens, Komis, Bashkirs, Mordvins, and others, in addition to 7 sub-populations of ethnic Russians (**Table 1**).

The uneven distribution of the genome data in Europe is further exacerbated by widespread lack of data sharing in the scientific community. While commercial companies are compiling but restricting massive data amongst themselves, the genome wide sequence data from ancestry testing and diagnostic sequencing [28] are difficult or impossible to retrieve [21,29]. This is usually justified by the possibility that, even in case when the databases are completely anonymous, it is technically impossible to identify participating individuals with additional genotype information [30]. Therefore, publishing individual genome data needs appropriate levels of informed consent that requires a combination of technical and societal stipulations within the context in which

the data are released. There remains a stunning lack of consensus or compelling legal precedents involving ownership or open release of Biomedical materials and derivative data [31]. Different published studies (genomes, GWAS, and others) have been collected under widely diverse levels of informed consent agreed by the study participants. All this ambiguity leads to resistance on the part of many in management/privacy positions to simply deny access to both samples and data, a serious problem that may be getting worse.

We emphasize the importance of the public access to the data, that is consented and open. The best practice, pioneered by G1K consortium, was to deposit the collected genome sequences, informed consent details, and the accompanying data into an international database that could serve as a valuable resource for the researchers worldwide, while providing security and protecting the interests of participating individuals and the communities they represent [4]. Publicly available genome data generated from the general population of the country has a vital role to unlock the capacities of genomic-based personal medicine for residents of a given county, and benefit everyone.

Abbreviations

HGP: Human Genome Project; G1K: 1000 Genomes Project; PCA: Principal Component Analysis; SNP: Single Nucleotide Polymorphism

Declarations

Ethics approval and consent to participate

This manuscript does not contain any individual data

Consent for publication

This manuscript does not contain any individual data

Data Availability

Links to all the data mentioned in this article are available in **Table S1**

Competing interests

The authors declare that they have no competing interests

Authors' contributions

TKO, has written the first draft. WWW prepared and analyzed the data, SM and SJO contributed to the original ideas and writing and final editing of the manuscript.

References

1. Lander ES, Linton LM, Birren B, Nusbaum C, Zody MC, Baldwin J, et al.. Initial sequencing and analysis of the human genome. *Nature*. 2001; doi: 10.1038/35057062.

2. Venter JC, Adams MD, Myers EW, Li PW, Mural RJ, Sutton GG, et al.. The Sequence of the Human Genome. *Science (1979)*. 2001; doi: 10.1126/science.1058040.

3. Cavalli -Sforza L. The Human Genome Diversity Project. *Special Meeting of UNSECO*. Paris: UESCO;

4. Auton A, Abecasis GR, Altshuler DM, Durbin RM, Bentley DR, Chakravarti A, et al.. A global reference for human genetic variation. *Nature*. 2015; doi: 10.1038/nature15393.

5. Smedley D, Smith KR, Martin A, Thomas EA, McDonagh EM, Cipriani V, et al.. 100,000 Genomes Pilot on Rare-Disease Diagnosis in Health Care — Preliminary Report. *New England Journal of Medicine*. 2021; doi: 10.1056/NEJMoa2035790.

6. Oleksyk TK, Brukhin V, O'Brien SJ. Putting Russia on the genome map. *Science (2015)*. 2015; doi: 10.1126/science.350.6262.747-a.

7. Mallick S, Li H, Lipson M, Mathieson I, Gymrek M, Racimo F, et al.. The Simons Genome Diversity Project: 300 genomes from 142 diverse populations. *Nature*. 2016; doi: 10.1038/nature18964.

8. Oleksyk TK, Wolfsberger WW, Weber AM, Shchubelka K, Oleksyk OT, Levchuk O, et al.. Genome diversity in Ukraine. *Gigascience*. 2021; doi: 10.1093/gigascience/giaa159.

9. Cavalli-Sforza LL, Menozzi P, Piazza A. The History and Geography of Human Genes. 1st ed. Princeton, NJ: Princeton University Press; ISBN:9780691187266

10. Seldin MF, Shigeta R, Villoslada P, Selmi C, Tuomilehto J, Silva G, et al.. European Population Substructure: Clustering of Northern and Southern Populations. *PLoS Genetics*. 2006; doi: 10.1371/journal.pgen.0020143.

11. Bauchet M, McEvoy B, Pearson LN, Quillen EE, Sarkisian T, Hovhannesyan K, et al.. Measuring European Population Stratification with Microarray Genotype Data. *The American Journal of Human Genetics*. 2007; doi: 10.1086/513477.

12. Novembre J, Johnson T, Bryc K, Kutalik Z, Boyko AR, Auton A, et al.. Genes mirror geography within Europe. *Nature*. 2008; doi: 10.1038/nature07331.

13. Khrunin A v., Khokhrin D v., Filippova IN, Esko T, Nelis M, Bebyakova NA, et al.. A Genome-Wide Analysis of Populations from European Russia Reveals a New Pole of Genetic Diversity in Northern Europe. *PLoS ONE*. 2013; doi: 10.1371/journal.pone.0058552.

14. Tambets K, Yunusbayev B, Hudjashov G, Ilumäe A-M, Rootsi S, Honkola T, et al.. Genes reveal traces of common recent demographic history for most of the Uralic-speaking populations. *Genome Biology*. 2018; doi: 10.1186/s13059-018-1522-1.

15. Avise JC. Phylogepgrpahy: The History and Formation of Species. Cambridge, MA: Harvard University Press; ISBN:9780674666382

16. Gudbjartsson DF, Helgason H, Gudjonsson SA, Zink F, Oddson A, Gylfason A, et al.. Large-scale whole-genome sequencing of the Icelandic population. *Nature Genetics*. 2015; doi: 10.1038/ng.3247.

17. Genomics England: The National Genomics Research and Healthcare Knowledgebase.

18. Oleksyk TK, Brukhin V, O'Brien SJ. The Genome Russia project: Closing the largest remaining omission on the world Genome map. *Gigascience*. 2015; doi: 10.1186/s13742-015-0095-0.

19. Ball MP, Bobe JR, Chou MF, Clegg T, Estep PW, Lunshof JE, et al.. Harvard Personal Genome Project: lessons from participatory public research. *Genome Medicine*. 2014; doi: 10.1186/gm527.

20. Smetana J, Brož P. National Genome Initiatives in Europe and the United Kingdom in the Era of Whole-Genome Sequencing: A Comprehensive Review. *Genes (Basel)*. 2022; doi: 10.3390/genes13030556.

21. Zhernakova DV, Brukhin V, Malov S, Oleksyk TK, Koepfli KP, Zhuk A, et al.. Genome-wide sequence analyses of ethnic populations across Russia. *Genomics*. 2020; doi: 10.1016/j.ygeno.2019.03.007.

22. Robinson WT, Fornell C. Sources of Market Pioneer Advantages in Consumer Goods Industries. *Journal of Marketing Research*. 1985; doi: 10.1177/002224378502200306.

23. Karczewski KJ, Francioli LC, Tiao G, Cummings BB, Alföldi J, Wang Q, et al.. The mutational constraint spectrum quantified from variation in 141,456 humans. *Nature*. 2020; doi: 10.1038/s41586-020-2308-7.

24. Gurdasani D, Carstensen T, Tekola-Ayele F, Pagani L, Tachmazidou I, Hatzikotoulas K, et al.. The African Genome Variation Project shapes medical genetics in Africa. *Nature*. 2015; doi: 10.1038/nature13997.

25. Kaja E, Lejman A, Sielski D, Sypniewski M, Gambin T, Suchocki T, et al.. 'The Thousand Polish Genomes Project' - a national database of Polish variant allele frequencies. *bioRxiv*. 2021; doi: 10.1101/2021.07.07.451425.

26. Altshuler DM, Durbin RM, Abecasis GR, Bentley DR, Chakravarti A, Clark AG, et al.. An integrated map of genetic variation from 1,092 human genomes. *Nature*. 2012; doi: 10.1038/nature11632.

27. Karow J. As Russia's War in Ukraine Continues, Genomics Research May Become Another Casualty. *GenomeWeb*. New York; 2022 Mar 28; https://www.genomeweb.com/genetic-research/russias-war-ukraine-continues-genomics-research-may-become-another-casualty

28. Laestadius LI, Rich JR, Auer PL. All your data (effectively) belong to us: data practices among direct-to-consumer genetic testing firms. *Genetics in Medicine*. 2017; doi: 10.1038/gim.2016.136.

29. Fakhro KA, Staudt MR, Ramstetter MD, Robay A, Malek JA, Badii R, et al.. The Qatar genome: a population-specific tool for precision medicine in the Middle East. *Human Genome Variation*. 2016; doi: 10.1038/hgv.2016.16.

30. Bonomi L, Huang Y, Ohno-Machado L. Privacy challenges and research opportunities for genomic data sharing. *Nature Genetics*. 2020; doi: 10.1038/s41588-020-0651-0.

31. O'Brien SJ. Stewardship of Human Biospecimens, DNA, Genotype, and Clinical Data in the GWAS Era. *Annual Review of Genomics and Human Genetics*. 2009; doi: 10.1146/annurev-genom-082908-150133.

Click here to access/download
Supplementary Material
Table S1.Data Links.xlsx
