# Supplementary material for: The Pioneer Advantage: Filling the blank spots on the map of genome diversity in Europe
Source: Gigascience. 2022 Sep 9;11:giac081. doi: 10.1093/gigascience/giac081 (PMC9463063; doi:10.1093/gigascience/giac081)
Supplement: giac081_Response_to_Reviewer_Comments_Original_Submission [file giac081_response_to_reviewer_comments_original_submission.pdf]

Author's Response To Reviewer Comments

Close

Dear Dr. Edmunds:

Thank you very much for the substantial review. We agreed with many of the statements, and made corrections where appropriate. We included two new paragraphs as requested in the MAJOR POINT, and responded to the minor issues in the point-by-point review below. We hope that you will find our responses satisfactory and looking forward to the positive decision regarding publication of our review.

Sincerely yours

Taras K. Oleksyk, Ph.D.
Department of Biological Sciences
Oakland University

MAJOR POINT:
What is missing is a brief discussion of gnomad and other projects that aggregate data to provide variant frequencies across different populations. The authors can provide some commentary on how these databases are useful in clinical variant interpretation and biomedical research, but what are the downfalls of not having access to all of the underlying data (for example bam files or gvcf files for each individual). Accessing datasets from dbGAP or different databases requires access permissions that can take a while to set up across many different projects and bioinformatics skills to process the data. It is useful for a wider audience if the data is in a web accessible format like gnomad database, but this type of database has its limitation"

ANSWER: Two paragraphs have been added to the manuscript on page 7 (lines 1-15) and page 10 (lines 19-29). The text is included below.

PARAGRAPH 1.
"Multiple types of efforts to aggregate current known variation are underway. To cover the highest number of samples and include projects with varying data release strategies, first the Exome Aggregation Consortium (ExAC) and then the Genome Aggregation Database (gnomAD) omitted the individual-level information. Today gnomAD and provided publicly available and accessible variation summaries from as many as 76,156 individual genomes of various projects across the globe [21,22]. These datasets are easy to handle and to interpret, and can be useful for comparative analysis and identification of novel variation, but not well suited for population analysis on the smaller scale. On the other hand, the database of Genotypes and Phenotypes (dbGaP) provide resources like the individual variants and extensive amount of phenotypical information per sample, but require additional bioinformatic expertise to handle [23]. Often, the nature of this data means that data access is controlled and requires an application with appropriate justification. To ensure data security, specialized instruments and tokens are often needed to reach the data. These necessary precautions can create delays or outright limit research group in their ability to reach the data, given lack of technical expertise."

PARAGRAPH 2.
"As stipulated in the original proposal and development documents for the Genome Russia and cooperative agreements for the Genome Diversity in Ukraine projects, the principal goal of these efforts was to provide open access of all sequence data SNP annotation and other genome features, so that these could join other international genome sequence consortia releasers [6,8,18,24]. The design and even the informed consent protocols for both of these projects were actually modeled after the 1,000 Genomes Project which had been vetted by the world's experts on human genome ethics. The original intention and promise was to join and to augment the catalogue of genomes in 1,000 Genomes Projects as well as the SNP annotations to complement ExAc[22], GnomAd [21], Gene Mutation Database

(HGMD) and HGMD-DM (disease-causing mutations) [31]. The political shuttering of open release by Russian authorities would ultimately cost all these important projects until release was remedied. This ill-advised suspension of open release was further exacerbated by the Feb 24, 2022 invasion of Ukraine ordered by Russian President Putin."

MINOR POINTS (point by point responce)

Page 1: "Currently, near ten thousand whole genome sequences from Europe are publicly available". There is much more available in gnomad so maybe specify the difference (Non-Finnish European 34,029, 5,316 Finnish)

Changed to: "Currently, near ten thousand whole genome sequences from Europe are publicly available".

Page 2: "geographic genome deserts fromm" typo should be "from"

corrected

Page 3: "medically related variants", change to "clinically relevant variants"

Corrected

Page 15: "Ukraine is an outlier on this category" should read "Ukraine is an outlier in this category"

Corrected

Page 14: "technically impossible", could this be "technically possible"

corrected

Page 18: "All this ambiguity leads to resistance on the part of many in management/privacy positions to simply deny access to both samples and data, a serious problem that may be getting worse." Not sure this is clear as written. I think it leads to simple deny of access, and not resistance to it.

Changed to: "All this ambiguity leads to resistance on the part of many".

Close
