# Supplementary material for: The Pioneer Advantage: Filling the blank spots on the map of genome diversity in Europe
Source: Gigascience. 2022 Sep 9;11:giac081. doi: 10.1093/gigascience/giac081 (PMC9463063; doi:10.1093/gigascience/giac081)
Supplement: giac081_Reviewer_1_Report_Original_Submission — Aniko Sabo -- 7/18/2022 Reviewed [file giac081_reviewer_1_report_original_submission.pdf]

Reviewer Report

Title: The Pioneer's Advantage: Filling the Blank Spots On the Map of Genome Diversity in Europe

Version: Original Submission **Date:** 7/18/2022

Reviewer name: Aniko Sabo

Reviewer Comments to Author:

This is a well written op-ed and review piece on uneven distribution of the available whole genome sequencing data in European populations. I only have one major and a few minor suggestions for revisions.
Major: What is missing is a brief discussion of gnomad and other projects that aggregate data to provide variant frequencies across different populations. The authors can provide some commentary on how these databases are useful in clinical variant interpretation and biomedical research, but what are the downfalls of not having access to all of the underlying data (for example bam files or gvcf files for each individual). Accessing datasets from dbGAP or different databases requires access permissions that can take a while to set up across many different projects and bioinformatics skills to process the data. It is useful for a wider audience if the data is in a web accessible format like gnomad database, but this type of database has its limitations.
Minor:
Page 1: "Currently, near ten thousand whole genome sequences from Europe are publicly available". There is much more available in gnomad so maybe specify the difference (Non-Finnish European 34,029, 5,316 Finnish)
Page 2: "geographic genome deserts fromm" typo should be "from"
Page 3: "medically related variants", change to "clinically relevant variants"
Page 15: "Ukraine is an outlier on this category" should read "Ukraine is an outlier in this category"
Page 14: "technically impossible", could this be "technically possible"
Page 18: "All this ambiguity leads to resistance on the part of many in management/privacy positions to simply deny access to both samples and data, a serious problem that may be getting worse." Not sure this is clear as written. I think it leads to simple deny of access, and not resistance to it.

Methods

Are the methods appropriate to the aims of the study, are they well described, and are necessary controls included? Choose an item.

Conclusions

Are the conclusions adequately supported by the data shown? Choose an item.

Reporting Standards

Does the manuscript adhere to the journal's guidelines on [minimum standards of reporting?] Choose an item.

Choose an item.

Statistics

Are you able to assess all statistics in the manuscript, including the appropriateness of statistical tests used? Choose an item.

Quality of Written English

Please indicate the quality of language in the manuscript: Choose an item.

Declaration of Competing Interests

Please complete a declaration of competing interests, considering the following questions:

- Have you in the past five years received reimbursements, fees, funding, or salary from an organisation that may in any way gain or lose financially from the publication of this manuscript, either now or in the future?
- Do you hold any stocks or shares in an organisation that may in any way gain or lose financially from the publication of this manuscript, either now or in the future?
- Do you hold or are you currently applying for any patents relating to the content of the manuscript?
- Have you received reimbursements, fees, funding, or salary from an organization that holds or has applied for patents relating to the content of the manuscript?
- Do you have any other financial competing interests?
- Do you have any non-financial competing interests in relation to this paper?

If you can answer no to all of the above, write 'I declare that I have no competing interests' below. If your reply is yes to any, please give details below.

I declare that I have no competing interests

I agree to the open peer review policy of the journal. I understand that my name will be included on my report to the authors and, if the manuscript is accepted for publication, my named report including any attachments I upload will be posted on the website along with the authors' responses. I agree for my report to be made available under an Open Access Creative Commons CC-BY license (http://creativecommons.org/licenses/by/4.0/). I understand that any comments which I do not wish to be included in my named report can be included as confidential comments to the editors, which will not be published.

Choose an item.

To further support our reviewers, we have joined with Publons, where you can gain additional credit to further highlight your hard work (see: https://publons.com/journal/530/gigascience). On publication of this paper, your review will be automatically added to Publons, you can then choose whether or not to claim your Publons credit. I understand this statement.

Yes Choose an item.
